# Intraplaque Enhancement Is Associated With Artery-to-Artery Embolism in Symptomatic Vertebrobasilar Atherosclerotic Diseases

**DOI:** 10.3389/fneur.2021.680827

**Published:** 2021-09-01

**Authors:** Zhikai Hou, Mingyao Li, Jinhao Lyu, Ziqi Xu, Yifan Liu, Jianfeng He, Jing Jing, Rong Wang, Yongjun Wang, Xin Lou, Zhongrong Miao, Ning Ma

**Affiliations:** ^1^Department of Interventional Neuroradiology, Beijing Tiantan Hospital, Capital Medical University, Beijing, China; ^2^China National Clinical Research Center for Neurological Diseases, Beijing, China; ^3^Department of Radiology, The First Medical Center of Chinese People's Liberation Army General Hospital, Beijing, China; ^4^Department of Neurology, The First Affiliated Hospital of College of Medicine, Zhejiang University, Hangzhou, China; ^5^Department of Neurology, Beijing Tiantan Hospital, Capital Medical University, Beijing, China; ^6^Tiantan Neuroimaging Center of Excellence, Beijing, China; ^7^Department of Neurosurgery, Beijing Tiantan Hospital, Capital Medical University, Beijing, China

**Keywords:** atherosclerosis, intracranial stenosis, vertebrobasilar disease, ischemic stroke, magnetic resonance imaging

## Abstract

**Objective:** There are limited data regarding the characteristics of intracranial plaques according to stroke mechanism in the posterior circulation. This study aims to compare whether the plaque characteristics and baseline features are different in patients with artery-to-artery (A-to-A) embolism and those with parent artery disease in the intracranial vertebrobasilar atherosclerotic disease.

**Methods:** From September 2014 to January 2017, patients with recent posterior circulation stroke due to intracranial vertebrobasilar atherosclerotic disease were retrospectively analyzed. Patients with the following eligibility criteria were included: (1) age ≥18 years old, (2) ischemic stroke in the vertebrobasilar territory, (3) 70–99% stenosis of the intracranial vertebral artery or basilar artery, and (4) two or more atherosclerotic risk factors. Patients with concomitant ipsilateral or bilateral extracranial vertebral artery >50% stenosis, cardio-embolism, or non-atherosclerotic stenosis were excluded. The plaque characteristics, including intraplaque compositions (intraplaque hemorrhage and intraplaque calcification), intraplaque enhancement, and remodeling index, were evaluated by using 3T high-resolution magnetic resonance imaging (HRMRI). The baseline features including vascular risk factors and the involved artery were collected. Patients were divided into A-to-A embolism and parent artery disease groups based on the diffusion-weighted images, T2-weighted images, or computed tomography. The plaque characteristics and baseline features were compared between the two groups.

**Results:** Among consecutive 298 patients, 51 patients were included. Twenty-nine patients had A-to-A embolism and 22 patients had parent artery disease. Compared with parent artery disease, the occurrence rates of intraplaque enhancement and intracranial vertebral involvement were higher in the A-to-A embolism group (79.3 vs. 36.4%; *p* = 0.002 and 62.1 vs. 18.2%; *p* = 0.002, respectively). Multivariable logistic regression analysis showed that intraplaque enhancement and intracranial vertebral artery plaques were also associated with A-to-A embolism (adjusted OR, 7.31; 95% CI 1.58–33.77; *p* = 0.011 and adjusted OR, 9.42; 95% CI 1.91–46.50; *p* = 0.006, respectively).

**Conclusion:** Intraplaque enhancement and intracranial vertebral artery plaques seem to be more closely associated with A-to-A embolism than parent artery disease in patients with symptomatic intracranial vertebrobasilar disease.

**Clinical Trial Registration:**http://www.clinicaltrials.gov, identifier: NCT02705599.

## Introduction

Posterior circulation stroke accounts for about 20–25% of ischemic stroke (1, 2), 9.9–16.3% of which is due to intracranial vertebrobasilar stenosis (3, 4). Patients with the symptomatic vertebrobasilar disease with evidence of impaired distal perfusion or blood flow due to severe stenosis are at a higher risk of recurrence with medical treatment (4–6). In these patients, the stroke mechanisms are usually impaired distal perfusion combined with A-to-A embolization or parent artery disease (7). Previous studies have shown that A-to-A embolism or multiple infarcts may be associated with vulnerable plaque, which has a higher risk of stroke recurrence (8–10). So far, studies on that whether the crucial plaque characteristics are different in patients with A-to-A embolism and those with parent artery disease due to severe intracranial vertebrobasilar stenosis are scarce.

Recently, HRMRI is increasingly used to understand the stroke mechanisms in patients with intracranial atherosclerotic stenosis. With the application of HRMRI, intracranial vessel wall affected by atherosclerosis can be imaged to display plaque components (11–13), plaque distribution (14, 15), and the degrees of enhancement after injection of Gadolinium-DTPA (16, 17). A few studies are unraveling the differences of the plaque features by using HRMRI between different stroke mechanisms in patients with anterior circulation stroke (18, 19), but is rarely studied in the posterior circulation.

Previous studies have shown that stroke mechanisms may differ in different intracranial artery territories (5, 20). The Warfarin–Aspirin Symptomatic Intracranial Disease study also showed that there was more parent artery disease in patients with posterior circulation stroke (35.9 vs. 15.3%) and less border-zone infarction (44.4 vs. 57.8%) than those in anterior circulation stroke (21). Furthermore, there are major differences in the mechanisms of stroke between the carotid and the middle cerebral arteries (5). However, a few studies focused on the difference between the intracranial vertebral and the basilar arteries.

The purpose of this study was aimed to explore the baseline features and the crucial plaque characteristics detected by HRMRI in severe intracranial vertebrobasilar stenosis and evaluate whether the plaque characteristics and the baseline features are different in patients with A-to-A embolism and those with parent artery disease.

## Materials and Methods

### Study Design and Subjects

This was a prospective, observational study approved by the ethics committees of Beijing Tiantan Hospital and Chinese PLA General Hospital. Written informed consent was obtained from the patients or their legal guardians.

From September 2014 to January 2017, we prospectively recruited ischemic stroke patients due to intracranial atherosclerotic stenosis. All patients received thorough evaluations to determine the ischemic stroke etiology, including computed tomography (CT), magnetic resonance imaging (MRI), magnetic resonance angiography (MRA), computed tomography angiography (CTA), or digital subtraction angiography (DSA), carotid Doppler ultrasonography, transcranial Doppler, echocardiography, electrocardiogram, etc. Patients with the following inclusion criteria were included: (1) age ≥18 years old, (2) ischemic stroke in the vertebrobasilar territory as identified by diffusion-weighted imaging (DWI)/T2-weighted imaging (T2WI)/CT, (3) 70–99% stenosis of the intracranial vertebral artery or basilar artery as confirmed by MRA, CTA, or DSA, and (4) two or more atherosclerotic risk factors including hypertension, hyperlipidemia, diabetes mellitus, obesity, and cigarette smoking. The definition of risk factor was the same as the previous protocol (22). Exclusion criteria were (1) coexistent >50% stenosis of the ipsilateral or bilateral extracranial vertebral artery; (2) evidence of cardio-embolism, such as atrial fibrillation, recent myocardial infarct within 4 weeks, mitral stenosis or prosthetic valve, etc.; (3) non-atherosclerotic vasculopathy that may result in ischemic stroke (e.g., vasculitis, moyamoya disease, or dissection).

Baseline features, including sex, age, vascular risk factors (hypertension, diabetes mellitus, hyperlipidemia, obesity, and cigarette smoking), previous stroke history, antithrombotic medication, and statin medication were recorded for each patient.

### Stroke Mechanisms Based on Routine Diffusion-Weighted Imaging/T2-Weighted Imaging/Computed Tomography and Magnetic Resonance Angiography/Computed Tomography Angiography/Digital Subtraction Angiography

All DWI/T2WI/CT and MRA/CTA/DSA images of patients were reviewed independently by two neurologists (MN and HZK) to determine the stroke mechanisms. In situations of disagreement, a third assessor adjudicated (XZQ). We classified the stroke mechanisms in the included patients as (1) parent artery atherosclerosis occluding penetrating artery (henceforth referred to as parent artery disease) is defined as isolated pons or medullary infarct on brain images, which localized in the territory of perforating arteries that arise at the site of the diseased basilar artery or vertebral artery. The parent artery was severe stenosed due to atherosclerosis. (2) A-to-A embolism is defined as single or multiple scattered cerebral or cerebellar cortical infarct(s) with or without subcortical infarcts (including the bilateral occipital lobe, medial temporal lobes, corpus callosum, midbrain, cerebellar hemisphere, and cerebellar vermis), which located in the branch territory of diseased intracranial vertebral artery or basilar artery. (23, 24).

### Plaque Features Identified by High-Resolution Magnetic Resonance Imaging

Details of the parameters of the HRMRI multiple sequences were the same as the previous studies (22, 25). The images were acquired by a coronal or axial view, which covered the intracranial vertebral artery and basilar artery. T1-weighted vessel wall images of responsible lesions were reconstructed into cross-sectional areas (perpendicular to the vessel longitudinal axis) at the image workstation. The evaluation was subsequently performed on the reformatted images using freely available software ImageJ (Rasband, National Institute of Mental Health, Bethesda, MD, USA) (22). All images were analyzed by two experienced neuroradiologists (LX and LJH) who were blinded to the clinical data of the patients. An image-quality rating (poor, adequate, and good) was given to each image by the two neuroradiologists. Poor-quality images with severe motion artifacts or low signal intensity-to-noise ratio were excluded. The present study did not display intra- and interobserver variabilities with the same or different scanners in light of previous research showing that these variabilities are small (25).

All atherosclerotic plaques that were involved in the intracranial vertebral artery or basilar artery on HRMRI were defined as eccentric wall thickening with or without luminal stenosis identified on the reconstructed precontrast HRMR images (26). A responsible vessel was the artery that supplied the infarcted tissues. A culprit plaque within the responsible vessel was the plaque if it was the only lesion within the culprit vascular territory of the stroke or the most stenotic lesion when multiple plaques were present (26). The plaque characteristics, including intraplaque compositions (intraplaque hemorrhage and intraplaque calcification), intraplaque enhancement, and remodeling index were assessed. Intraplaque hemorrhage was defined as the brightest plaque signal intensity >150% of that of the adjacent gray matter on T1 sequences (12). Intra-plaque calcification was defined as a low-signal intensity spot in the plaque on all pulse sequences (27). The mean signal intensity values of culprit plaques and the normal adjacent vessel wall were measured on precontrast and postcontrast HRMR images. Intraplaque enhancement was defined as the enhancement degree of the culprit plaque higher than that of the normal adjacent vessel wall (26). The vessel areas at the maximal lumen narrowing site and reference site were automatically calculated after the manual vessel contour tracing in the software ImageJ (Rasband, National Institute of Mental Health, Bethesda, MD, USA). The remodeling index was defined and calculated as the ratio of the vessel area at the maximal lumen narrowing site to that at the reference site. The reference site was selected based on the WASID (Warfarin–Aspirin Symptomatic Intracranial Disease) method (28). Remodeling index ≥1.05 was defined as positive remodeling, 0.95 and 1.05 as intermediate remodeling, and ≤ 0.95 as negative remodeling (29).

### Statistical Analysis

Continuous variables are presented as mean and standard deviation (SD) or median with interquartile range (IQR); categorical variables are presented as percentages. Categorical variables were analyzed using a χ2 test or Fisher's exact test. Continuous variables were compared using Student's *t*-test between the two groups. The associations between plaque characteristics and stroke mechanisms were further assessed by using multivariable logistic regression models. Variables were included for multivariate analysis if they were *p* ≤ 0.2 in the univariate analysis. The adjusted odds ratio (OR) and their 95% confidence intervals (CI) were calculated. A two-sided *p*-value of <0.05 indicated statistical significance. All statistical analyzes were performed using commercial software (SPSS).

## Results

### The Baseline Features of the Patients

**Figure 1:**
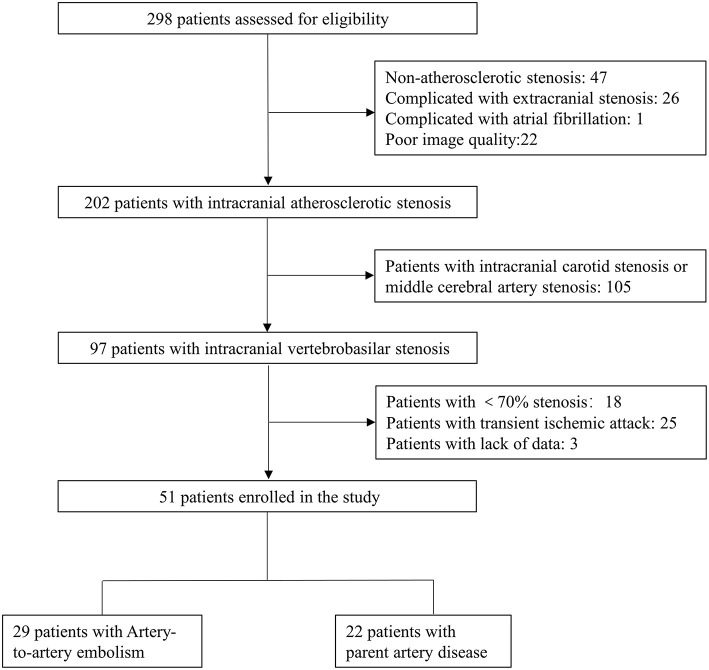
Flow chart of the study.

The flow chart of the study is presented in Figure 1. Among 298 consecutive patients, 51 patients fulfilled the inclusion criteria. Of the 51 patients, 29 (56.9%) were determined as A-to-A embolism and 22 (43.1%) as parent artery disease. Detailed infarct distributions and MRA/CTA/DSA of included patients are presented in Supplementary Figure 1. Patient demographics, atherosclerotic risk factors, and the culprit plaque characteristics on HRMRI are presented in Table 1. The median time from the qualifying event to HRMRI was 31 days (IQR, 14–48 days).

**Table 1:** Baseline and plaque characteristics.

**Characteristics**	**Values**
Age (mean ± SD, years)	58.0 ± 8.82
Male sex, *n* (%)	44/51 (86.3%)
**Risk factors**	
Hypertension, *n* (%)	44/51 (86.3%)
Diabetes mellitus, *n* (%)	22/51 (43.1%)
Hyperlipidemia, *n* (%)	21/51 (41.2%)
Obesity, *n* (%)	19/51 (37.3%)
Cigarette smoking, *n* (%)	35/51 (68.6%)
Ischemic stroke history, *n* (%)	12/51 (23.5%)
Antithrombotic medication, *n* (%)	6/51 (11.8%)
Statin medication, *n* (%)	10/51 (19.6%)
Time from qualifying event to HRMRI, median (IQR), days	31 (14–48)
**Stroke mechanisms**	
Parent artery disease, *n* (%)	22/51 (43.1%)
A-to-A embolism, *n* (%)	29/51 (56.9%)
**Location of culprit plaque**	
BA, *n* (%)	29/51 (56.9%)
Intracranial VA, *n* (%)	22/51 (43.1%)
**Plaque compositions**	
Intraplaque hemorrhage, *n* (%)	12/51 (23.5%)
Intraplaque calcification, *n* (%)	4/51 (7.8%)
**Plaque enhancement degree**	
Non-enhancement, *n* (%)	20/51 (39.2%)
Intraplaque enhancement, *n* (%)	31/51 (60.8%)
Remodeling index (mean ± SD)	1.10 ± 0.332

*HRMRI, high-resolution magnetic resonance imaging; A-to-A embolism, artery-to-artery embolism; BA, basilar artery; VA, vertebral artery*.

### Comparisons of the Baseline and Plaque Features of the Two Groups

There were no significant differences found between the two groups in sex, age, atherosclerotic risk factors, or time from the qualifying event to HRMRI. Previous stroke history, antithrombotic medication, and statin medication had no significant difference between the A-to-A embolism group and the parent artery disease group (Table 2).

**Table 2:** Patient baseline characteristics between the parent artery disease group and A-to-A embolism group.

**Clinical characteristics**	**Parent artery disease (*n* = 22)**	**A-to-A embolism (*n* = 29)**	***p*-value**
Age (mean ± SD, years)	56.2 ± 8.33	59.3 ± 9.09	0.208
Male sex, *n* (%)	17/22 (77.3%)	27/29 (93.1%)	0.216
**Risk factors**			
Hypertension, *n* (%)	20/22 (90.9%)	24/29 (82.8%)	0.684
Diabetes mellitus, *n* (%)	10/22 (45.5%)	12/29 (41.4%)	0.771
Hyperlipidemia, *n* (%)	10/22 (45.5%)	11/29 (37.9%)	0.589
Obesity, *n* (%)	9/22 (40.9%)	10/29 (34.5%)	0.638
Cigarette smoking, *n* (%)	14/22 (63.6%)	21/29 (72.4%)	0.503
Ischemic stroke history, *n* (%)	7/22 (31.8%)	5/29 (17.2%)	0.224
Antithrombotic medication, *n* (%)	4/22 (18.2%)	2/29 (6.9%)	0.383
Statin medication, *n* (%)	5/22 (22.7%)	5/29 (17.2%)	0.894
Time from qualifying event to HRMRI, median (IQR), days	30.5 (14.8–35.5)	31 (13–50)	0.700

*HRMRI, high-resolution magnetic resonance imaging; A-to-A embolism, artery-to-artery embolism*.

**Figure 2:**
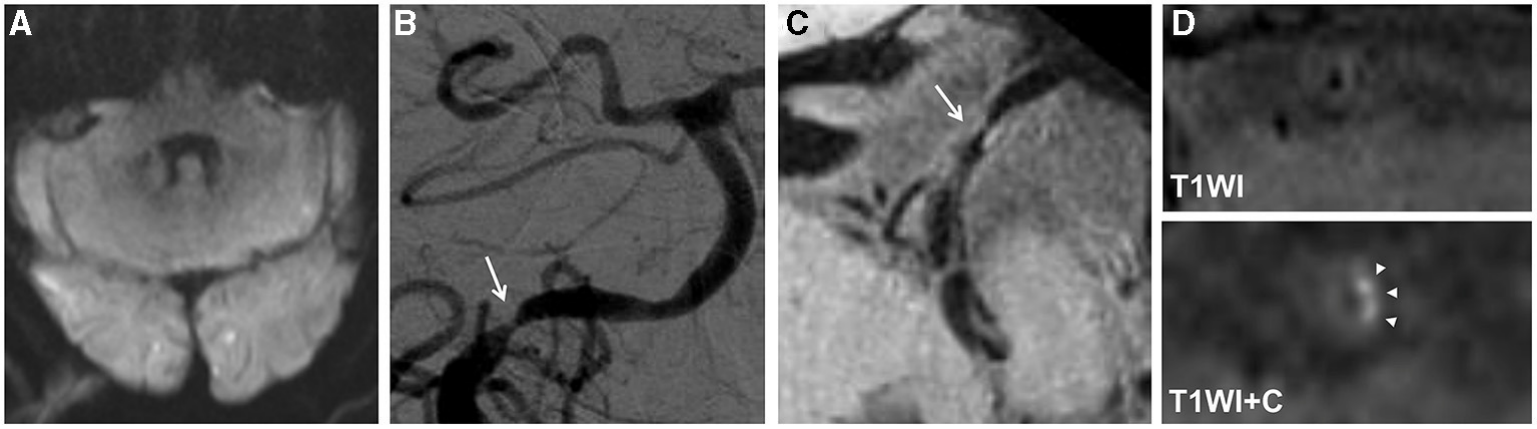
An 80-year-old man presented with dizziness for 15 days. The diffusion-weighted imaging (DWI) showed infarcts of bilateral occipital lobes and left cerebellum **(A)**. The digital subtraction angiography (DSA) showed severe stenosis at the V4 segment of the right vertebral artery (arrow) **(B)**. The high-resolution magnetic resonance imaging (HRMRI) demonstrated a diffuse distributive plaque (arrow) **(C)** with an eccentric enhancement (arrowheads) on T1-weighted image after gadolinium–DTPA injection **(D)**.

**Figure 3:**
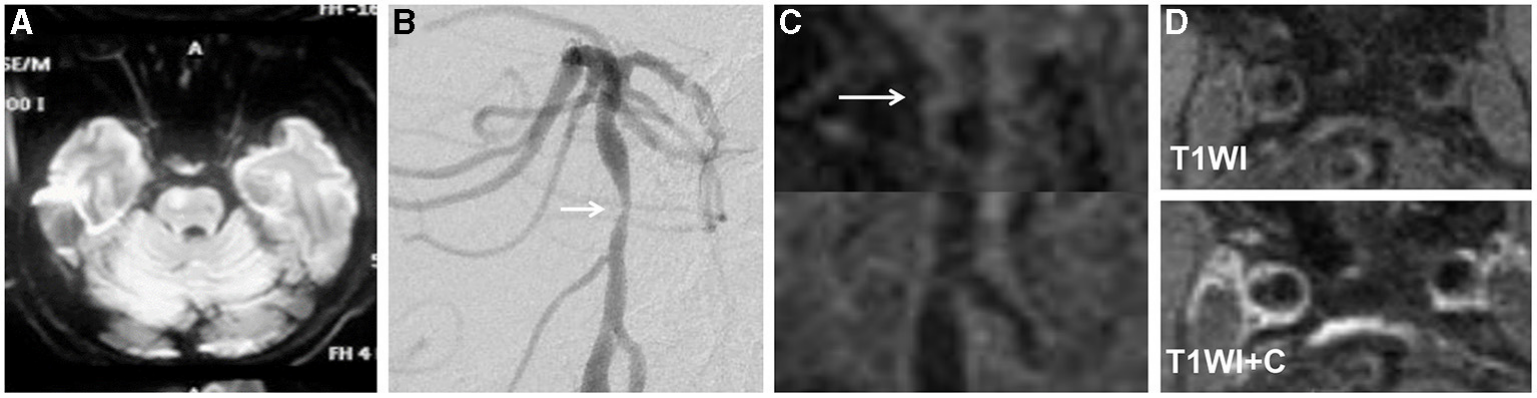
A 68-year-old man presented with dysarthria for 20 days. The DWI showed an infarct located at the right pons **(A)**. The DSA showed severe stenosis in the middle segment of the basilar artery (arrow) **(B)**. The HRMRI demonstrated a diffuse distributive plaque (arrow) **(C)**. There was no plaque enhancement on T1-weighted image after gadolinium–DTPA injection **(D)**.

In the A-to-A embolism group, the occurrence rates of intracranial vertebral artery involvement and intraplaque enhancement were higher than those in the parent artery disease group (62.1 vs. 18.2%; *p* = 0.002 and 79.3 vs. 36.4%; *p* = 0.002) (Table 3; Figures 2, 3). Culprit plaque was found in 81.8% of the basilar artery in the parent artery disease compared with 37.9% of the basilar artery in the A-to-A embolism group (*p* = 0.002). No significant difference was found between the two groups in plaque compositions and remodeling index.

**Table 3:** Comparison of culprit plaque characteristics between the parent artery disease group and the A-to-A embolism group.

**HRMRI characteristics**	**Univariable**			**Multivariable**	
	**Parent artery disease (*n* = 22)**	**A-to-A embolism (*n* = 29)**	***p*-value**	**Adjusted OR (95% CI)**	***p*-value**
**Location of culprit plaque**			0.002	–	–
BA, *n* (%)	18/22 (81.8%)	11/29 (37.9%)	–	Ref	–
Intracranial VA, *n* (%)	4/22 (18.2%)	18/29 (62.1%)	–	9.42 (1.91–46.50)	0.006
**Plaque compositions**					
Intraplaque hemorrhage, *n* (%)	3/22 (13.6%)	9/29 (31.0%)	0.147	–	–
Intraplaque calcification, *n* (%)	1/22 (4.5%)	3/29 (10.3%)	0.625	–	–
**Enhancement degree**			0.002	–	–
Non-enhancement, *n* (%)	14/22 (63.6%)	6/29 (20.7%)	–	Ref	–
Intraplaque enhancement, *n* (%)	8/22 (36.4%)	23/29 (79.3%)	–	7.31 (1.58–33.77)	0.011
Remodeling index (mean ± SD)	1.08 ± 0.373	1.12 ± 0.304	0.672	–	–

*HRMRI, high-resolution magnetic resonance imaging; A-to-A embolism, artery-to-artery embolism; BA, basilar artery; VA, vertebral artery*.

Location of culprit plaque, intraplaque hemorrhage, and enhancement degree were used as input variables for the multivariable logistic regression analysis. After adjusting for confounding factors, intraplaque enhancement was associated with A-to-A embolism (adjusted OR, 7.31; 95% CI 1.58–33.77; *p* = 0.011). The intracranial VA of culprit plaque was also associated with A-to-A embolism (adjusted OR, 9.42; 95% CI 1.91–46.50; *p* = 0.006) (Table 3).

## Discussion

In patients with symptomatic severe intracranial vertebrobasilar atherosclerosis, we found that the patients with A-to-A embolism were more likely to have a higher occurrence of intraplaque enhancement and a higher rate of intracranial vertebral artery involvement than those with parent artery disease. The findings of our study have not previously been well-established in the literature.

Previous studies have indicated that there was an association between intraplaque enhancement and symptomatic intracranial atherosclerotic diseases. These studies found that plaque enhancement is more commonly observed in symptomatic intracranial artery stenosis (17, 30). Qiao et al. found that contrast enhancement of an intracranial plaque is associated with its likelihood to have caused a recent ischemic event, regardless of the plaque thickness (26). A previous study showed that intraplaque enhancement was independently associated with stroke recurrence (hazard ratio: 7.42, 95% CI: 1.74–31.75, *p* = 0.007) (16). However, a few studies of intracranial vessel wall imaging have concentrated on the relationship between stroke mechanisms and intraplaque enhancement. The result of our study showed that intraplaque enhancement was higher in the A-to-A embolism than in the parent artery disease. A similar finding was reported in a previous study focusing on evaluating the relationship between the middle cerebral artery plaque features on HRMRI and the stroke mechanism (19). In this study, patients with A-to-A embolism were more likely to have a high occurrence of plaque enhancement than non-A-to-A embolism (75.0 vs. 21.4%, *p* = 0.02) (19). We think the plaque enhancement may be more related to the A-to-A embolism than the other stroke mechanisms in symptomatic intracranial atherosclerosis.

Plaque enhancement related to plaque vulnerability has been reported in several previous studies on coronary and carotid artery atherosclerosis (31–33). Studies on HRMRI findings of the internal carotid artery and its pathologic specimen from endarterectomy showed that enhanced plaque is associated with abundant active inflammatory cells, neo-vessel formation, and fibrous cap thinning (34). The exact mechanism of intracranial plaque enhancement remains unclear due to the relative inaccessibility of specimens. An analogous process may occur in the intracranial vasculature in the setting of ICAD. The instability of ICAD plaques secondary to neovascularization and inflammation can present with thromboembolism events, leading to multiple cerebral infarcts. When the fibrous cap of vulnerable plaque ruptures, plaque content and mural thrombus drop into the distal territory of the downstream vessel and result in A-to-A embolism. Focal plaque or thrombus occluding the perforator ostium caused parent artery disease. Considering the patients with A-to-A embolism have a higher recurrent stroke rate than stroke of other mechanisms (10), the plaques causing A-to-A embolism may be more vulnerable than the plaques causing parent artery disease. The intraplaque enhancement may be used as an imaging biomarker to predict the risk of recurrent stroke. This needs to be confirmed in future studies.

Furthermore, we have found that intracranial atherosclerotic stroke mechanisms differed between the intracranial vertebral artery and the basilar artery. The A-to-A embolism was higher in the intracranial vertebral artery stenosis than that in basilar artery stenosis, whereas parent artery disease was higher in basilar artery stenosis than in the intracranial vertebral stenosis. This difference may be attributable to more perforating vessels arising from the basilar artery that may be more vulnerable to occlusion in the presence of parental artery plaque. There are multiple groups of perforating vessels arising from the basilar artery, such as paramedian arteries, short lateral circumferential arteries, and long lateral circumferential arteries. In the New England Medical Center Posterior Circulation Registry, distal territory embolism accounts for 32.0% among patients with the symptomatic intracranial vertebral disease (35). A Korea prospective multicenter study also reported that A-to-A embolism was most frequent in the intracranial vertebral artery (53%), whereas parent artery disease was most frequently associated with the basilar artery (64%) (5). Our findings were similar to those published in previous studies.

In our study, there was no significant difference in the occurrence of intraplaque hemorrhage between A-to-A embolism and parent artery disease. As far as intraplaque hemorrhage, a previous study reported that hyperintense plaques on T1-weighted images were more closely associated with A-to-A embolism than non-A-to-A embolism (75.0 vs. 21.1%, *p* < 0.001) in the anterior circulation (18). This finding was not found in the present study. The occurrence rate of intraplaque hemorrhage was higher in this study than in our study [47.3% (35/74) vs. 23.5% (12/51)]. The following two factors may explain the difference. The first was the intrinsic difference between the anterior circulation and posterior circulation. The second was the changes in the signal of intraplaque hemorrhage over time. The duration of intraplaque hemorrhage signal on HRMRI in the cerebral arteries has not been established. Longer time from symptom onset to HRMRI scanning in our study (median time 31 days) was observed compared with that in the previous study (mean time 8.3 days).

In our study, no significant differences in other plaque features including intraplaque calcification and remodeling index were found between A-to-A embolism and parent artery disease. Intraplaque calcification and remodeling patterns (positive remodeling) reflected the features of advanced plaque, but there were no differences found between the A-to-A embolism and parent artery disease in posterior circulation stroke. These findings need to be confirmed in future studies.

There are several limitations to this study. First, the sample size was small, and it may lead to type II errors. Only patients with severe vertebrobasilar stenosis were included, and this may cause inclusion criteria bias. In addition, there are three patients with basilar artery stenosis with pontine infarction and more than two embolic infarctions in the posterior cerebral artery territory in this study. The stroke mechanism is usually regarded as the combined mechanism. Considering the small sample size, we divided them into the A-to-A embolism group. Large sample size studies are needed to compare the plaque characteristic among the combined mechanism, A-to-A embolism, and parent artery disease. Second, the previous study showed that the intracranial plaque may be enhanced within weeks to months of cerebral infarction (36), but the median time from qualifying event to HRMRI was 31 (IQR, 14–48) days in this study. Intracranial plaque enhancement might be attenuated as the interval from qualifying event to HRMRI scan prolonged. This may result in biased results. The relationship between intraplaque enhancement and interval between qualifying event to image scan should be explored in the future study. Third, all patients are Chinese, and the result may not generalize the other ethnic population. Fourth, the majority of patients with A-to-A embolism underwent intracranial angioplasty and stenting, so it was impossible to compare the long-term outcome in variable stroke mechanisms groups.

## Conclusion

In patients with symptomatic intracranial vertebrobasilar stenosis, A-to-A embolism seems to be more closely associated with intraplaque enhancement and intracranial vertebral artery plaques than parent artery disease. Further studies are needed to confirm these findings.

## Data Availability Statement

The original contributions presented in the study are included in the article/Supplementary Material, further inquiries can be directed to the corresponding author.

## Ethics Statement

The studies involving human participants were reviewed and approved by Beijing Tantian Hospital and Chinese PLA General Hospital. The patients/participants provided their written informed consent to participate in this study. Written informed consent was obtained from the individual(s) for the publication of any potentially identifiable images or data included in this article.

## Author Contributions

ZH and NM: study concept and design, data analysis, drafting the manuscript, and full responsibility of data. ML, YL, and JH: data collection. JL, ZX, JJ, and XL: imaging data analysis. RW, YW, XL, and ZM: study concept and design of the work. All authors approved the final version to be published and they agreed to be accountable for all aspects of the work in ensuring that questions related to the accuracy or integrity of any part of the manuscript are appropriately investigated and resolved.

## Conflict of Interest

The authors declare that the research was conducted in the absence of any commercial or financial relationships that could be construed as a potential conflict of interest.

## Publisher's Note

All claims expressed in this article are solely those of the authors and do not necessarily represent those of their affiliated organizations, or those of the publisher, the editors and the reviewers. Any product that may be evaluated in this article, or claim that may be made by its manufacturer, is not guaranteed or endorsed by the publisher.
