# Supplementary material for: Intraplaque Enhancement Is Associated With Artery-to-Artery Embolism in Symptomatic Vertebrobasilar Atherosclerotic Diseases
Source: Front Neurol. 2021 Sep 1;12:680827. doi: 10.3389/fneur.2021.680827 (PMC8440987; doi:10.3389/fneur.2021.680827)
Supplement: Supplementary file 1 [file Data_Sheet_1.PDF]

SUPPLEMENTAL MATERIAL

Figure e-1. Detail infarct distributions and MRA/CTA/DSA of enrolled patients.

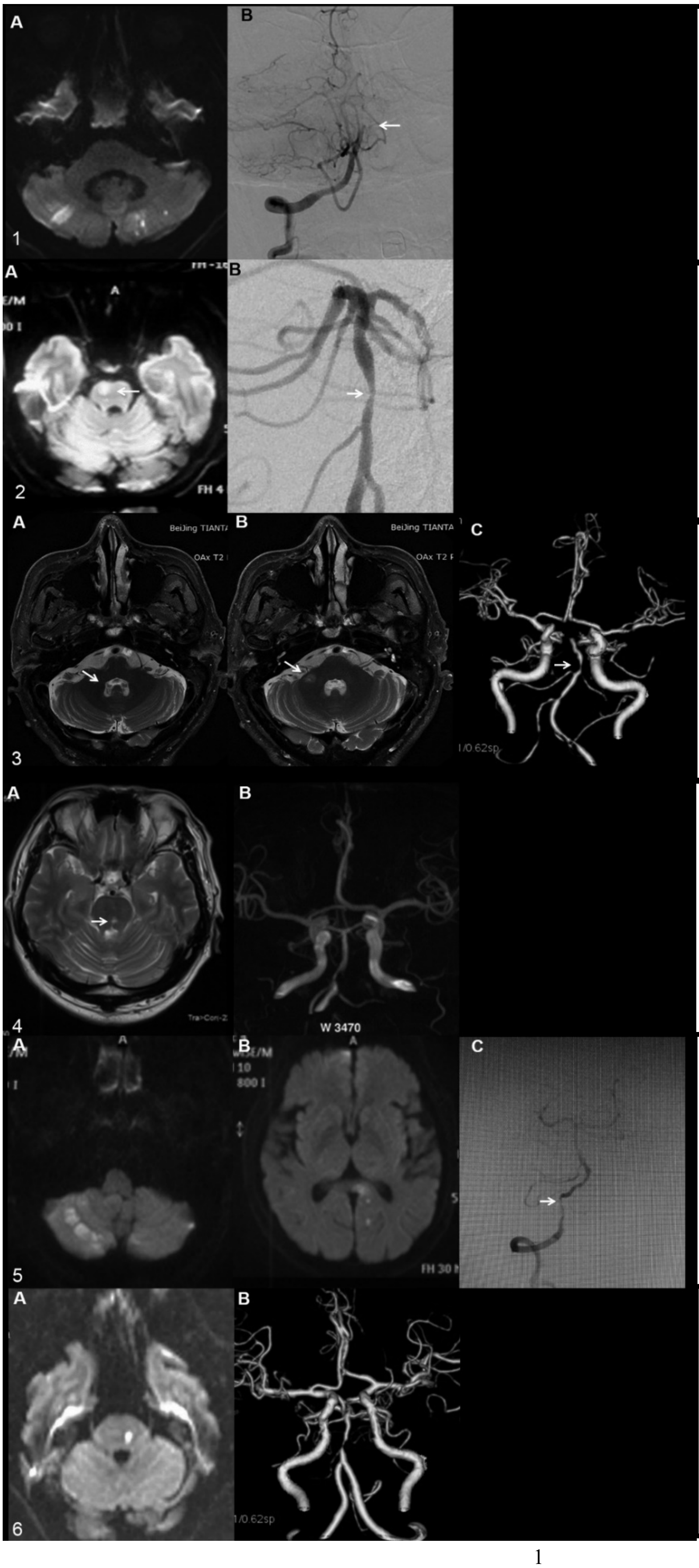

Age/Gender: 61/Male
Infarct distribution: Bilateral cerebellums
Responsible vessel: RV4
Stroke mechanisms:
Artery-to-artery embolism

68/Male
Right pons
BA
Perforator stroke

55/Male
Right brachium pontis
BA
Perforator stroke

53/Male
Left pons
BA
Perforator stroke

69/Male
Right cerebellum, left occipital lobe and left splenium of the corpus callosum
RV4
Artery-to-artery embolism

56/Male
Left pons
BA
Perforator stroke

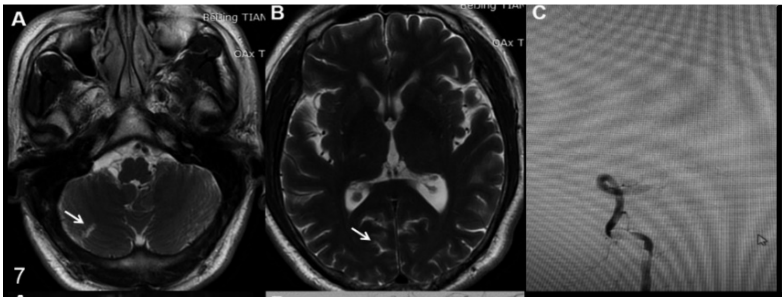	53/Male<br>Right cerebellum and bilateral occipital lobes<br>RV4<br>Artery-to-artery embolism
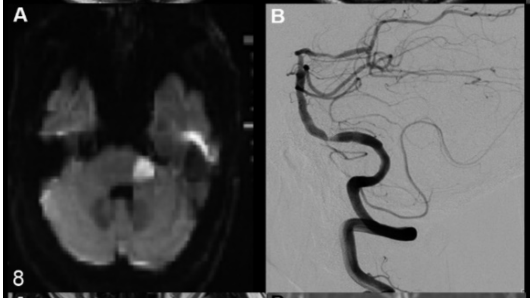	62/Male<br>Left brachium pontis<br>BA<br>Perforator stroke
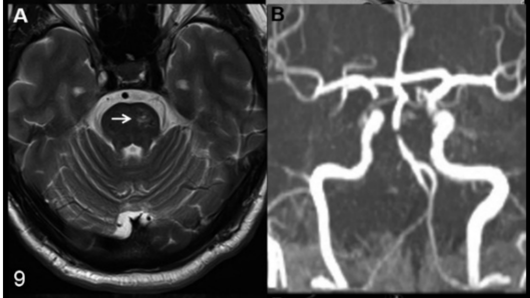	55/Male<br>Left pons<br>BA<br>Perforator stroke
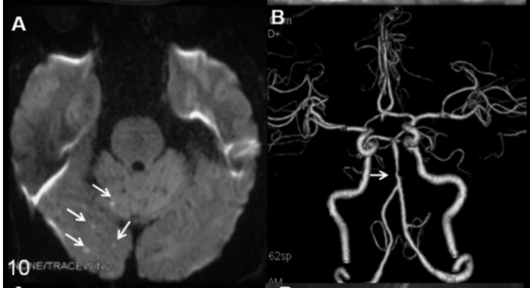	62/Male<br>Right occipital lobe and right cerebellar vermis<br>BA<br>Artery-to-artery embolism
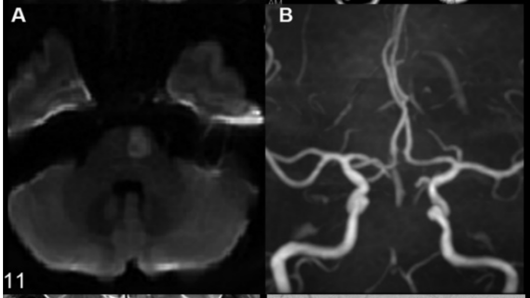	36/Female<br>Left pons<br>BA<br>Perforator stroke
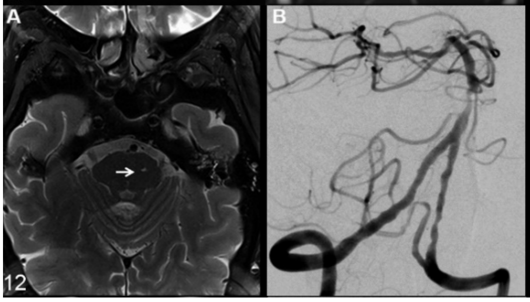	61/Male<br>Left pons<br>BA<br>Perforator stroke

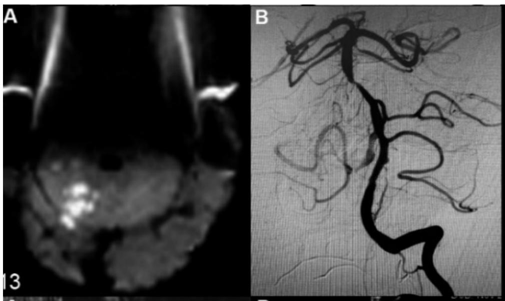

66/Male

Right cerebellar vermis

BA

Artery-to-artery embolism

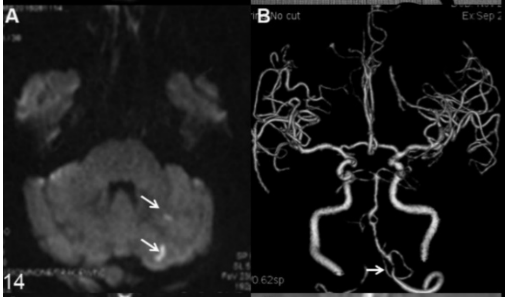

63/Male

Left cerebellum

LV4

Artery-to-artery embolism

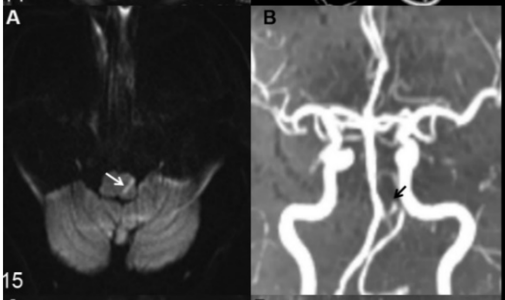

52/Female

Left medulla

LV4

Perforator stroke

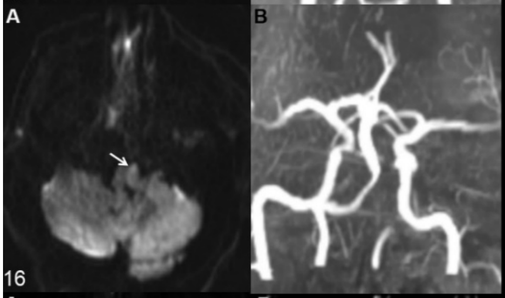

54/Male

Left medulla

LV4

Perforator stroke

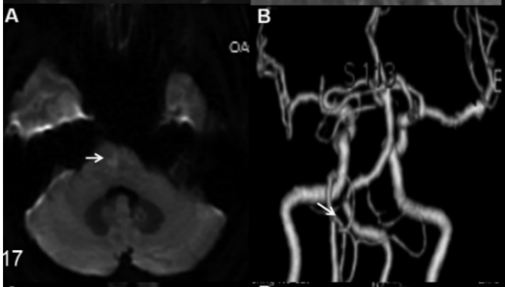

54/Male

Right pons

BA

Perforator stroke

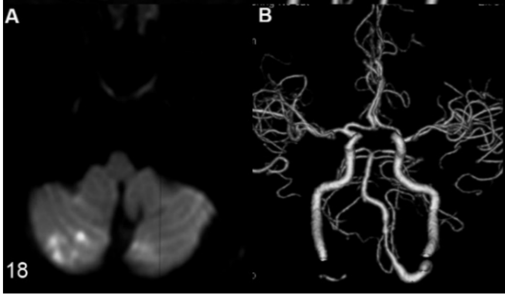

64/Male

Right cerebellum

RV4

Artery-to-artery embolism

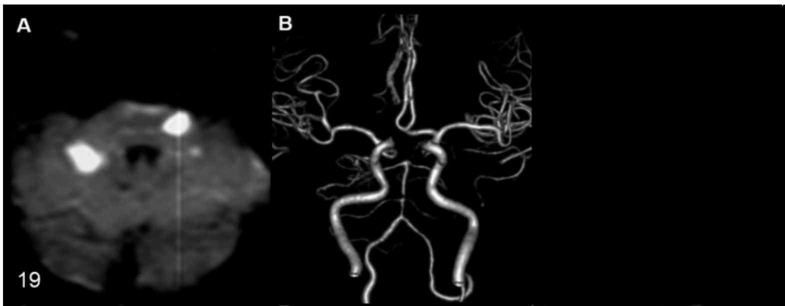

74/Female

Bilateral brachium pontis

BA

Artery-to-artery embolism

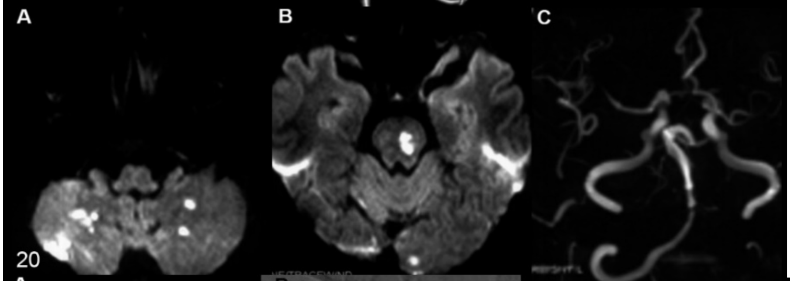

70/Male

Left pons, bilateral cerebellum, and right occipital lobe

RV4

Artery-to-artery embolism

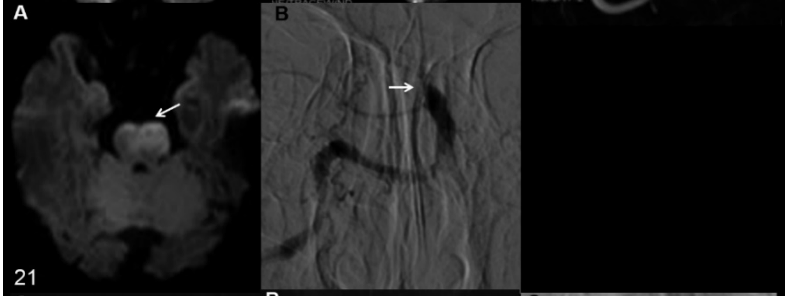

52/Male

Left pons

BA

Perforator stroke

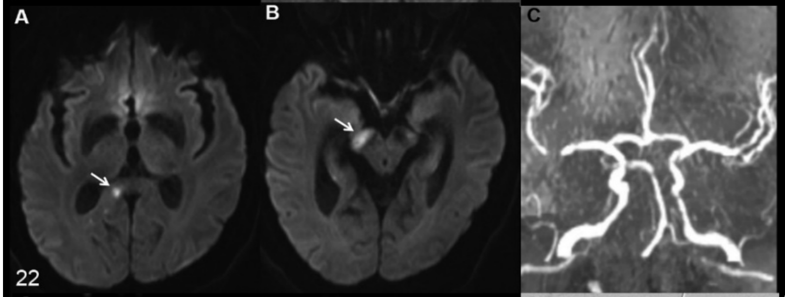

58/Male

Right brachium pontis, midbrain and right splenium of the corpus callosum

BA

Artery-to-artery embolism

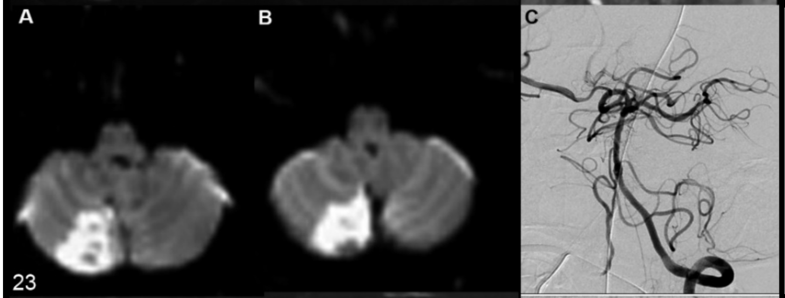

53/Male

Right cerebellum

RV4

Artery-to-artery embolism

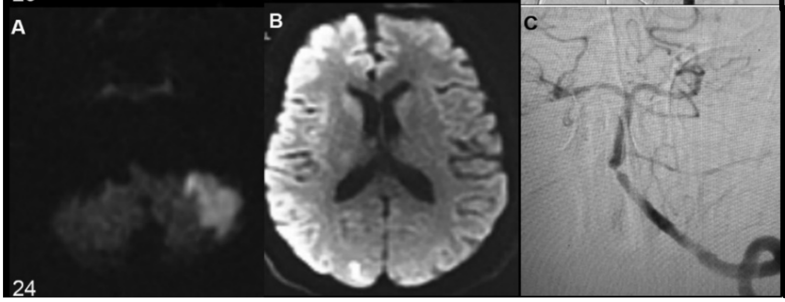

57/Male

left cerebellum and bilateral occipital lobe

BA

Artery-to-artery embolism

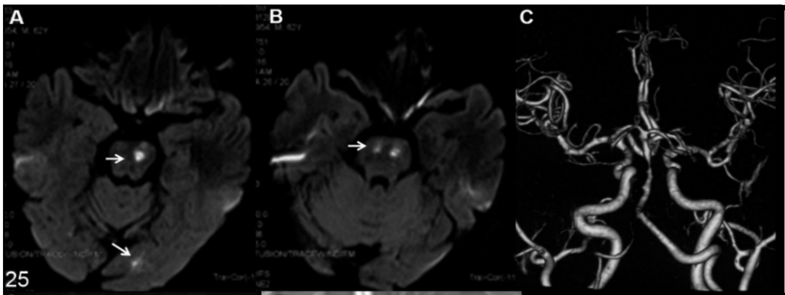

62/Male
Bilateral pons, right thalamus and left occipital lobe
BA
Artery-to-artery embolism

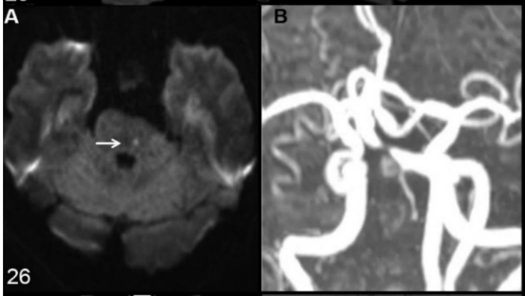

55/Male
Right pons
BA
Perforator stroke

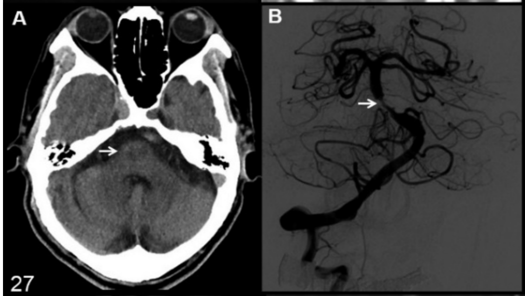

71/Male
Left pons
BA
Perforator stroke

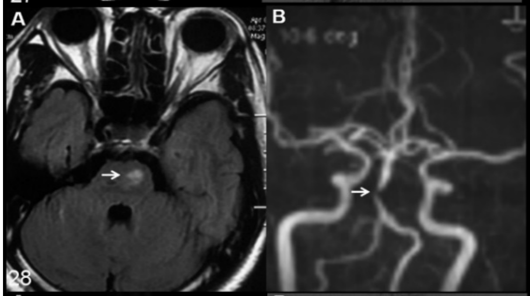

57/Male
Left pons
BA
Perforator stroke

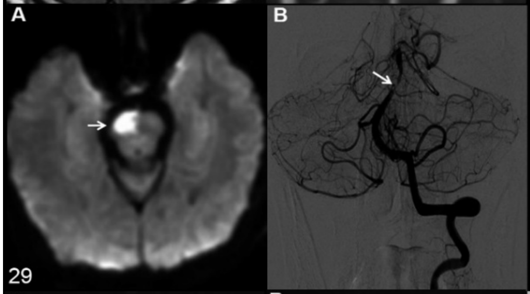

62/Female
Right pons
BA
Perforator stroke

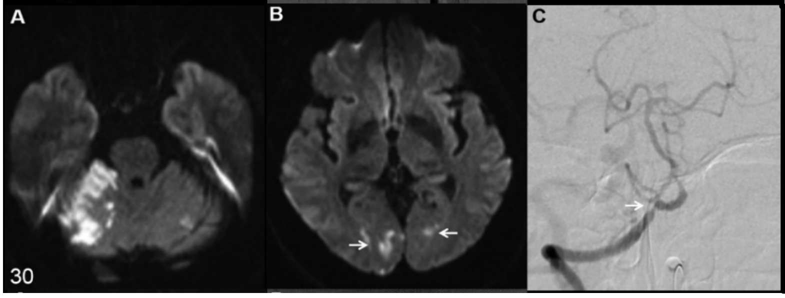

60/Male
Bilateral cerebellum and occipital lobes
RV4
Artery-to-artery embolism

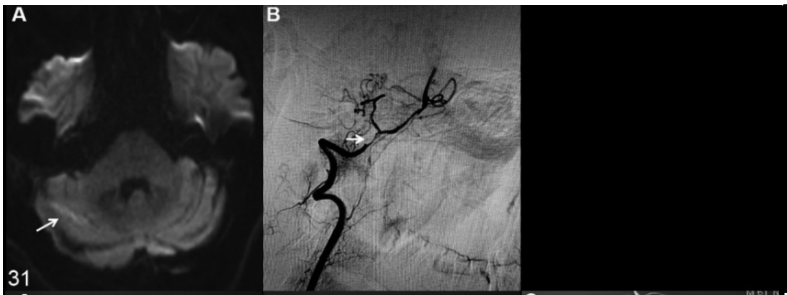

41/Male

Right cerebellum

RV4

Artery-to-artery embolism

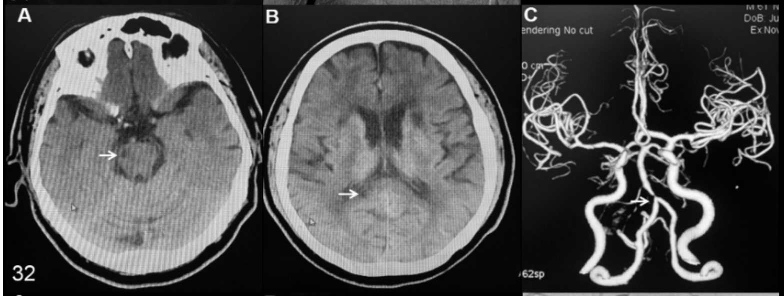

61/Male

Pons, left midbrain, and splenium of the corpus callosum

BA

Artery-to-artery embolism

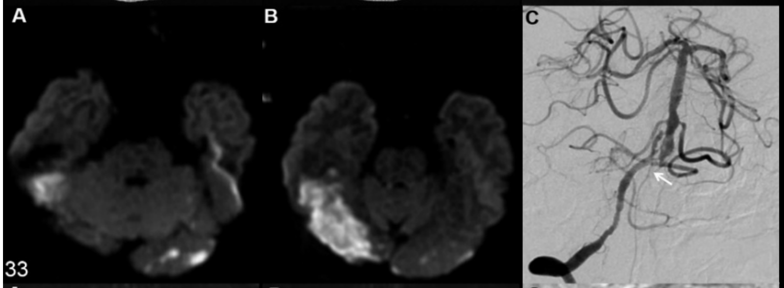

62/Male

Bilateral cerebellums and right occipital lobe

RV4

Artery-to-artery embolism

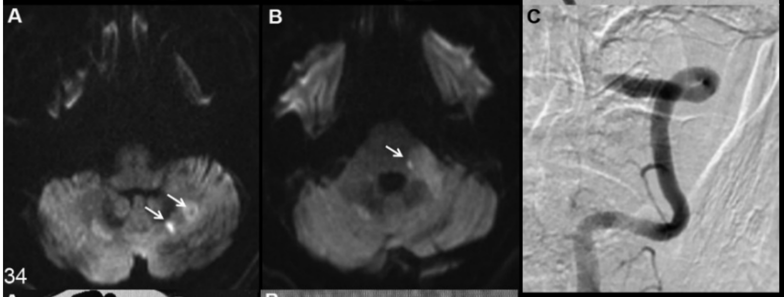

65/Male

Left cerebellum and brachium pontis

LV4

Artery-to-artery embolism

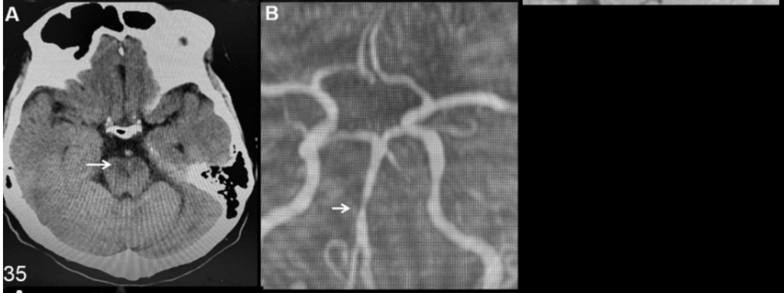

50/Male

Left pons

BA

Perforator stroke

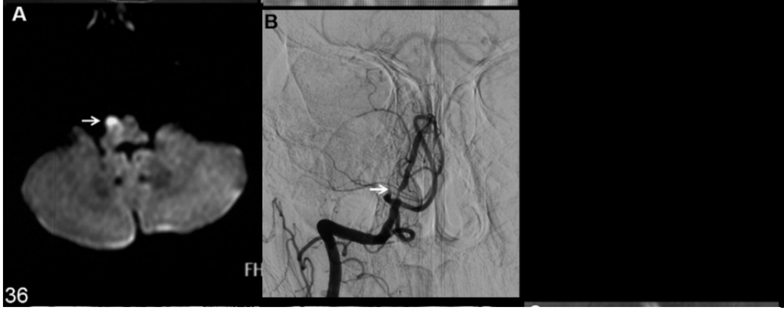

52/Male

Right medulla

RV4

Perforator stroke

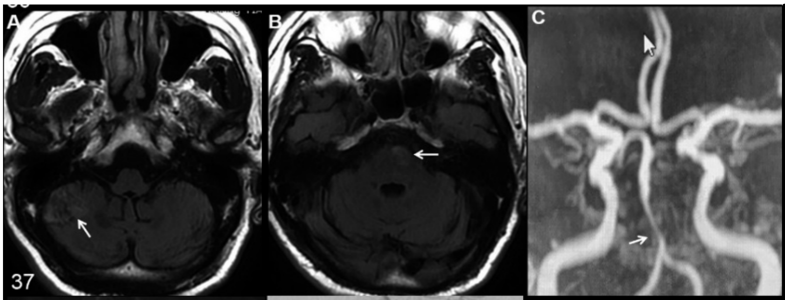

54/Male

Left pons and right cerebellum

BA

Artery-to-artery embolism

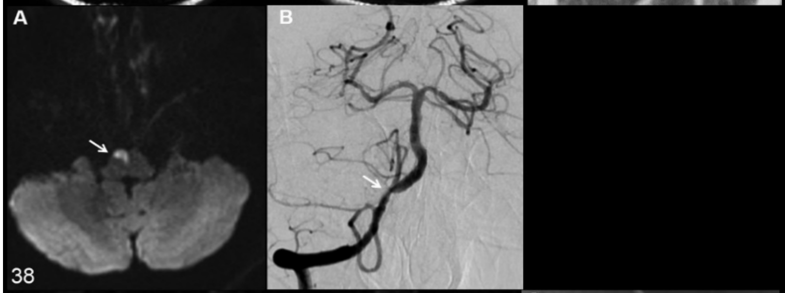

47/Male

Right medulla

RV4

Perforator stroke

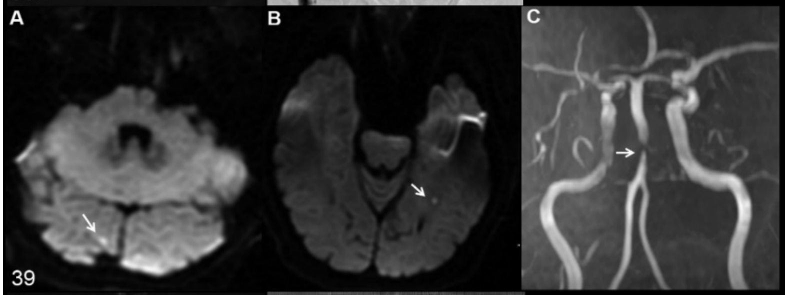

62/Male

Left occipital lobe

BA

Artery-to-artery embolism

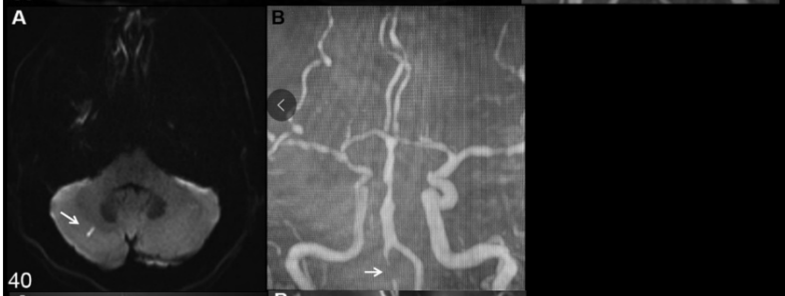

50/Male

Right cerebellum

RV4

Artery-to-artery embolism

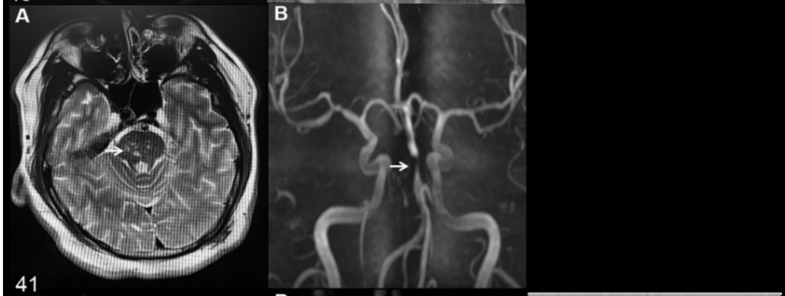

47/Male

Left pons

BA

Perforator stroke

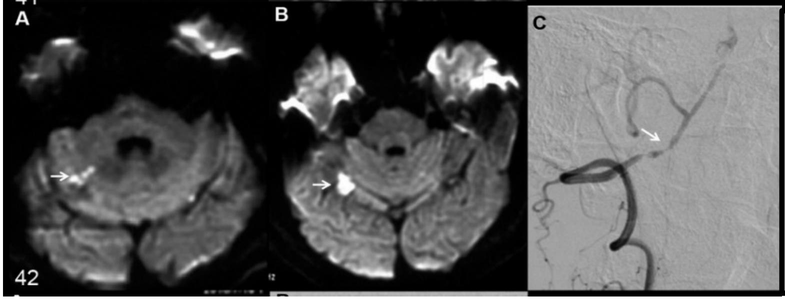

64/Male

Right cerebellum

RV4

Artery-to-artery embolism

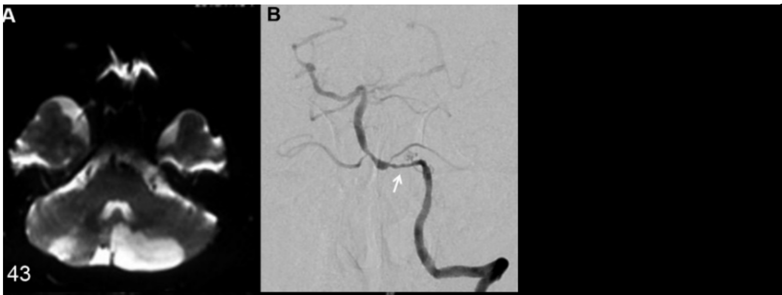

42/Male

Bilateral cerebellums

LV4

Artery-to-artery embolism

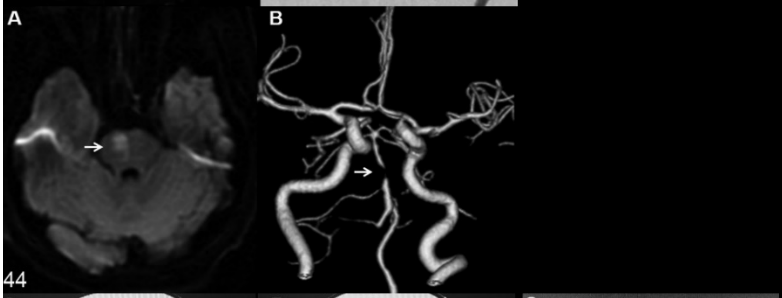

66/Male

Left pons

BA

Perforator stroke

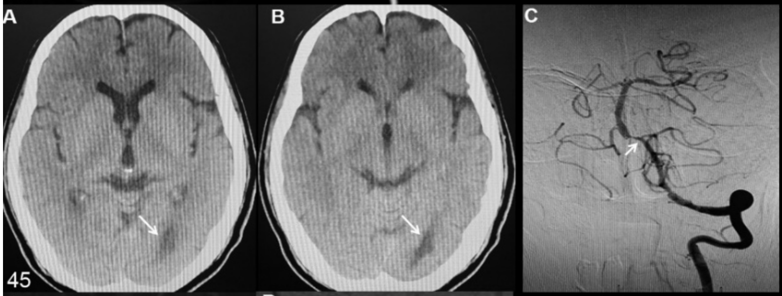

63/Male

Left occipital lobe

LV4

Artery-to-artery embolism

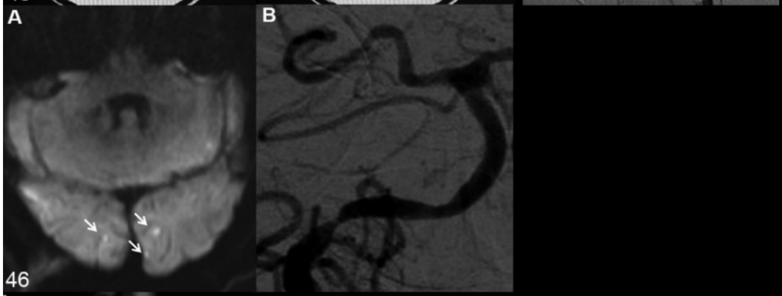

80/Male

Bilateral occipital lobes

RV4

Artery-to-artery embolism

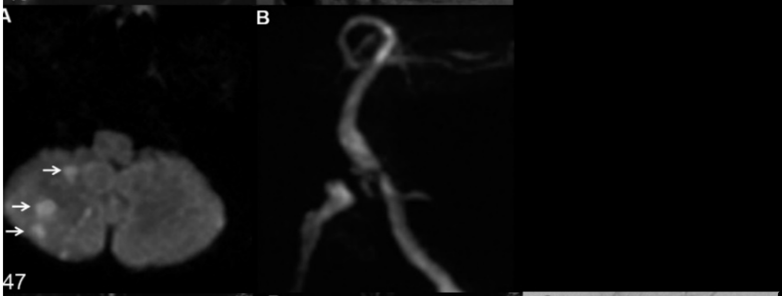

52/Male

Right cerebellum

RV4

Artery-to-artery embolism

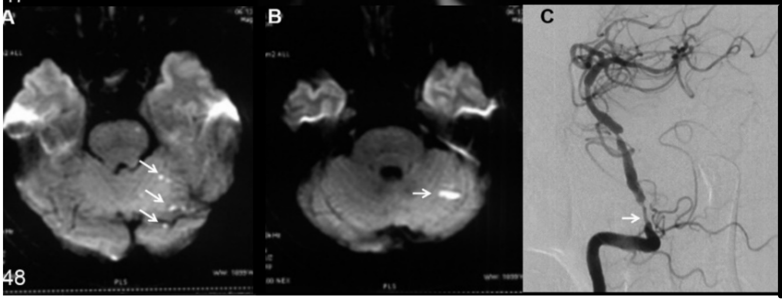

52/Male

Left cerebellum and occipital lobe

LV4

Artery-to-artery embolism

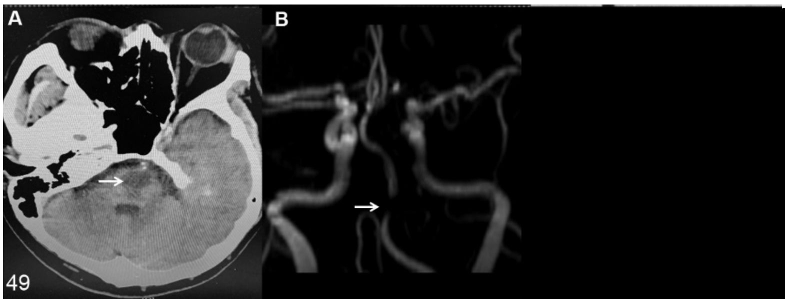	71/Female<br>Right pons<br>BA<br>Perforator stroke
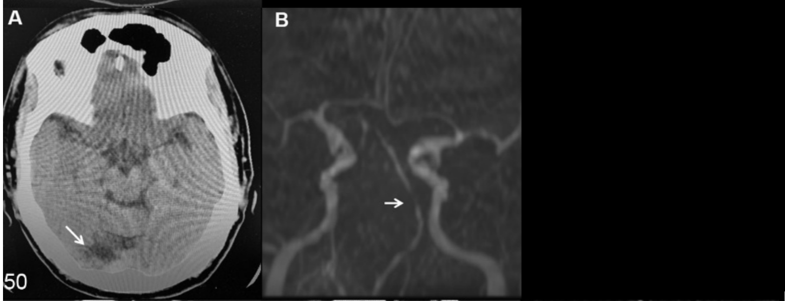	59/Male<br>The right occipital lobe, pons, and cerebellar vermis<br>BA<br>Artery-to-artery embolism
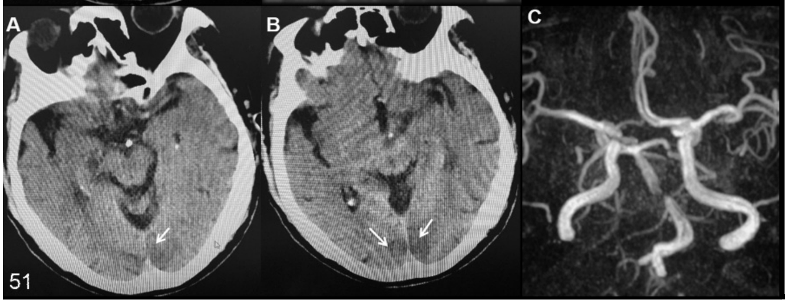	41/Male<br>bilateral occipital lobes<br>BA<br>Artery-to-artery embolism

Abbreviations: BA, basilar artery; V4, the V4 segment of vertebral artery.
